# Health Information Seeking Behaviors on Social Media During the COVID-19 Pandemic Among American Social Networking Site Users: Survey Study

**DOI:** 10.2196/29802

**Published:** 2021-06-11

**Authors:** Stephen Neely, Christina Eldredge, Ron Sanders

**Affiliations:** 1 School of Public Affairs College of Arts and Sciences University of South Florida Tampa, FL United States; 2 School of Information College of Arts and Sciences University of South Florida Tampa, FL United States; 3 Florida Center for Cybersecurity, School of Information College of Arts and Sciences University of South Florida Tampa, FL United States

**Keywords:** social media, internet, communication, public health, COVID-19, usage, United States, information seeking, web-based health information, survey, mistrust

## Abstract

**Background:**

In recent years, medical journals have emphasized the increasingly critical role that social media plays in the dissemination of public health information and disease prevention guidelines. However, platforms such as Facebook and Twitter continue to pose unique challenges for clinical health care providers and public health officials alike. In order to effectively communicate during public health emergencies, such as the COVID-19 pandemic, it is increasingly critical for health care providers and public health officials to understand how patients gather health-related information on the internet and adjudicate the merits of such information.

**Objective:**

With that goal in mind, we conducted a survey of 1003 US-based adults to better understand how health consumers have used social media to learn and stay informed about the COVID-19 pandemic, the extent to which they have relied on credible scientific information sources, and how they have gone about fact-checking pandemic-related information.

**Methods:**

A web-based survey was conducted with a sample that was purchased through an industry-leading market research provider. The results were reported with a 95% confidence level and a margin of error of 3. Participants included 1003 US-based adults (aged ≥18 years). Participants were selected via a stratified quota sampling approach to ensure that the sample was representative of the US population. Balanced quotas were determined (by region of the country) for gender, age, race, and ethnicity.

**Results:**

The results showed a heavy reliance on social media during the COVID-19 pandemic; more than three-quarters of respondents (762/1003, 76%) reported that they have relied on social media at least “a little,” and 59.2% (594/1003) of respondents indicated that they read information about COVID-19 on social media at least once per week. According to the findings, most social media users (638/1003, 63.6%) were unlikely to fact-check what they see on the internet with a health professional, despite the high levels of mistrust in the accuracy of COVID-19–related information on social media. We also found a greater likelihood of undergoing vaccination among those following more credible scientific sources on social media during the pandemic (*χ*^2^_16_=50.790; *φ*=0.258; *P*<.001).

**Conclusions:**

The findings suggest that health professionals will need to be both strategic and proactive when engaging with health consumers on social media if they hope to counteract the deleterious effects of misinformation and disinformation. Effective training, institutional support, and proactive collaboration can help health professionals adapt to the evolving patterns of health information seeking.

## Introduction

In recent years, medical journals have emphasized the increasingly critical role that social networking sites (SNSs) play in the dissemination of public health information and disease prevention guidelines [1,2]. Still, platforms such as Facebook and Twitter continue to pose unique challenges for clinical health care providers and public health officials alike. Although the public has grown more reliant on social media to stay informed during times of crisis [3], the information they receive comes from a variety of sources that are not always official or objective in nature. Health professionals often lack the time and resources that are necessary to keep pace with the rapidity of these web-based information environments. Moreover, effective health communications are increasingly complicated by factors such as politicization, antiscientific sentiments, and the potential that social networks have in rapidly spreading false information [4-6]. These challenges are perhaps the most acute under crisis conditions, which place unique strains on both health care systems and traditional information networks [7,8].

Although these concerns extend beyond the COVID-19 pandemic, their urgency has been underscored by the ongoing crisis. From the outset of the pandemic, public health officials noted an alarming volume of erroneous misinformation (as well as malicious disinformation) associated with COVID-19 on social media. Estimates from early studies have suggested that as much as 25% of the COVID-19–related information circulating on platforms like Twitter may contain some degree of misinformation [9]. The World Health Organization labeled this phenomenon as an “infodemic” and suggested that such misinformation can “lead to poor observance of public health measures, thus reducing their effectiveness and endangering countries’ ability to stop the pandemic” [10].

In order to effectively communicate during public health emergencies, it is increasingly critical for health professionals to understand how patients gather health-related information on the internet and adjudicate the merits of such information. However, while much has been written about social media’s expanding role in health communications, very little empirical data have been collected to examine how the public actually uses social media to learn and stay informed about ongoing health emergencies. To that end, we conducted a survey of 1003 American adults in order to better understand how heavily they have relied on social media and the specific ways in which they have used social media to learn about the COVID-19 pandemic. In light of the growing concerns over the proliferation of misinformation on social media, this study also aims to aid both health care practitioners and researchers in understanding how SNS users interact with and rely on credible scientific sources and how they have gone about fact-checking pandemic-related information. Collectively, this study seeks to better inform health communications through an enriched understanding of social media’s evolving role in health information seeking.

## Methods

A web-based survey of 1003 US-based adults was conducted (January 9 to January 12, 2021) through Prodege MR, an industry-leading market research provider. This survey was funded by Florida’s Center for Cybersecurity at the University of South Florida. Survey respondents were selected by using a stratified quota sampling approach to ensure that the sample was representative. Balanced quotas were determined (by region of the country) for gender, age, race, ethnicity, and education based on the US Census Bureau’s 2019 American Community Survey (ACS). Table 1 provides a summary of the comparison between sample respondents and the 2019 ACS data.

The initial sampling target was a total of 1067 individuals (which represents a margin of error 3.0 for the US population). However, data cleaning revealed a small number of incomplete and unusable responses, resulting in a total sample size of 1003 (a margin of error 3.09 for the US population). The results were reported with a 95% confidence level and a margin of error of 3.09. It is worth noting that the data collection method necessarily excluded those who lack internet access. However, given that the focus of the study was on social media users, this did not represent a serious threat to validity. Perhaps more importantly, the method naturally underrepresented those with lower levels of education. We made deliberate attempts to target this group during survey administration, though a gap persisted for those lacking a high school diploma (Table 1).

**Table 1:** Sample comparison.^a^

Characteristic		Sample in this study, %	2019 American Community Survey, %
**Gender**			
	Female	51.2	51.1
	Male	48.8	48.9
**Age (years)**			
	18-24	11.3	11.9
	25-34	17.8	17.8
	35-44	17	16.5
	45-54	16	16
	55-64	17.1	16.6
	≥65	20.8	21.2
**Race**			
	Black or African American	15.7	15.1
	White	73.2	76.4
	Asian or Pacific Islander	7.9	7.6
	American Indian or Alaska Native	1	0.8
	Other	2.2	0.2
**Ethnicity**			
	Hispanic	17.7	17.5
	Non-Hispanic	82.3	82.5
**Education**			
	Less than high school	6.8	11.4
	High school or equivalent	25.9	27.6
	Some college or an associate degree	34	30.4
	4-year degree	21.5	19.3
	Graduate or professional degree	11.7	11.4

^a^Data are from the Florida Center for Cybersecurity’s January 2021 COVID-19 survey.

## Results

### Summary of Results

Although the growing importance of social media in health communications has been widely discussed, our understanding of these trends, particularly those at the consumer level, has been largely anecdotal. The results reported below add some empirical context to our general understanding of these trends while also contributing important new data to enhance our understanding of how SNS users seek out and fact-check medical information during a public health emergency. The results are reported in subsections that follow the organization of the survey.

### Reliance and Confidence

The results from the survey affirm that Americans have relied heavily on social media to stay informed about COVID-19. Among the survey respondents, more than three-quarters (762/1003, 76%) stated that they have relied on social media at least “a little” to stay informed about the COVID-19 pandemic, while just under half (458/1003, 45.6%) reported that they have relied on social media “a lot” (Table 2). Further, 59.2% (594/1003) of respondents indicated that they read information about COVID-19 on social media at least once per week, while roughly one-third (323/1003, 32.2%) do so every day. These responses highlight the extent to which social media has become a primary source of health information for Americans; a large number of users (762/1003, 76%) reported that they do not merely encounter such content on the internet but also rely on platforms such as Facebook and Twitter for health information.

**Table 2:** Reliance on social media for pandemic-related information.^a^

Questions and responses			Respondents, n (%)
**How much have you relied on social media to stay informed about the COVID-19 pandemic?**			
	A great deal	208 (20.7)	
	A lot	250 (24.9)	
	A little	304 (30.3)	
	Not at all	241 (24)	
**On average, how often do you read information about COVID-19 on social media?**			
	Every day	323 (32.2)	
	A few days a week	271 (27)	
	Once a week	112 (11.2)	
	Less often	297 (29.6)	
**I am confident in the accuracy of the information I see about COVID-19 on social media**			
	Strongly agree	73 (7.3)	
	Somewhat agree	250 (24.9)	
	Neither agree nor disagree	252 (25.1)	
	Somewhat disagree	188 (18.7)	
	Strongly disagree	240 (23.9)	

^a^Data are from the Florida Center for Cybersecurity’s January 2021 COVID-19 survey.

Although people’s reliance on SNSs has been remarkably high throughout the pandemic, only about one-third of respondents (323/1003, 32.2%) expressed confidence in the accuracy of the information that they receive about COVID-19 on social media. Although paradoxical, this finding is consistent with prior studies that have shown a similar lack of confidence in the accuracy of news and political information on the internet, despite the increased reliance on such web-based media for information seeking [11]. In our study, this may, at least in part, reflect the sharp politicization of the COVID-19 pandemic [12]. Among the survey respondents, more three-quarters (761/1003, 76.1%) agreed that “politics has made it harder to learn the truth about Covid-19.” For health professionals, this highlights the extent to which the politicization of public health efforts can obscure scientific guidance and complicate health communications, especially when treatment and mitigation become matters of public policy.

### Fact-checking Social Media

It has been recently been noted in medical literature that identifying and mitigating misinformation on social media is a growing priority for health professionals [4]. It has also been pointed out that doing so will require proactive steps, such as “meeting people where they are and through the information networks and devices they use for day-to-day interactions” [13]. With these goals in mind, it is important for health professionals to understand the propensity of social media users to validate and verify (ie, fact-check) potential misinformation that they encounter on the internet and to understand the types of sources that social media users are turning to for guidance during public health crises. Prior research has suggested that rigorous fact-checking is less common on social media [14], and the survey responses appear to affirm this suggestion. Only about one-third of respondents (365/1003, 36.4%) indicated that they have “talked to a doctor or healthcare professional about the accuracy of something they saw on social media related to Covid-19” (Table 3). In contrast, respondents were almost twice as likely to have “talked to friends, family, or coworkers” about such information (686/1003, 68.4%), and 22% of respondents were more likely to have conducted their own internet research (578/1003, 57.6%) to fact-check COVID-19–related information on social media.

**Table 3:** Fact-checking pandemic-related information on social media.^a^

Responses to the following question: “Please indicate whether or not you've done each of the following since the start of the pandemic?”	Respondents, n (%)
“…done internet research to fact-check something that I saw on social media related to COVID-19”	578 (57.6)
“…talked to friends, family, or coworkers about the accuracy of something I saw on social media related to COVID-19”	686 (68.4)
“…talked to my doctor or a healthcare professional about the accuracy of something that I saw on social media related to COVID-19”	365 (36.4)

^a^Data are from the Florida Center for Cybersecurity’s January 2021 COVID-19 survey.

### Following Scientific Sources

**Figure 1:**
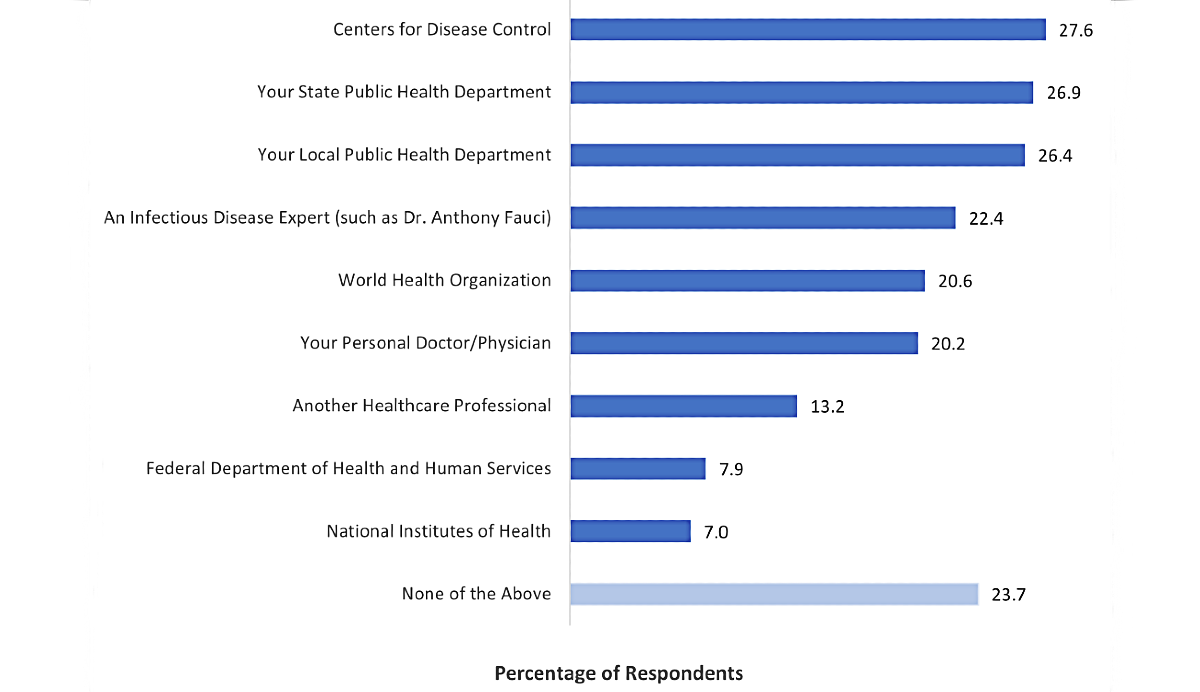
Health Information Sources "Followed" (As % of Respondents).

Although only about one-third of respondents reported actively fact-checking information with a medical professional, 581/762 (76.2%) reported that they followed at least 1 authoritative scientific source on social media during the pandemic (Figure 1). The data in Figure 1 only represent those who reported at least “a little” reliance on social media to stay informed about COVID-19 (n=762). More than one-quarter of respondents began following the Centers for Disease Control (210/762, 27.6%), their state public health department (205/762, 26.9%), or their local public health department (201/762, 26.4%). Just under one-quarter of respondents reported following an infectious disease expert such as Dr Anthony Fauci (171/762, 22.4%). Further, one-fifth of respondents (154/762, 20.2%) reported following their own personal doctor or physician, while 101/762 (13.2%) began following “another healthcare professional.”

Perhaps surprisingly, the data showed that respondents were slightly more likely to have followed institutional actors on social media (ie, the Centers for Disease Control and Prevention or a public health department) than they were to have followed individual health experts (ie, their personal physician or an infectious disease expert). This is somewhat inconsistent with findings from prior research, which have suggested that individual actors are typically more influential on social media [15,16]. The nature and severity of the COVID-19 pandemic may account for this difference, though additional research into this topic is warranted in order to better inform professional best practices.

On one hand, the responses were promising in the sense that most social media users did appear to have intentionally expanded their web-based networks during the pandemic to include credible institutional and individual medical and scientific sources (ie, 581/762 [76.2%] have followed at least 1 authoritative scientific source). On the other hand, it was noteworthy that nearly one-quarter of those who relied on social media for pandemic-related information (181/762, 23.8%) did not choose to follow such sources, and only one-fifth of respondents (154/762, 20.2%) began engaging with their own personal physician (ie, the health care provider whom they are most likely to trust for personalized health guidance) on social media.

### Scientific Credibility and Vaccine Intentions

As previous literature has suggested, the dissemination of credible scientific information on social media is necessary for disease prevention and effective public health management. The crosstabs from our survey highlighted one specific example of this; those who opted to follow credible scientific sources were significantly more likely to indicate that they will accept a COVID-19 vaccine (*P*<.001). When asked, 588/1003 (58.6%) of respondents to the survey reported that they would definitely or probably undergo vaccination [17]. Those who followed at least 2 credible public health or medical sources were 10% more likely to indicate that they would “definitely get vaccinated” than those who did not follow any such sources (Table 4). Those who followed 4 or more such sources were over 25% more likely to report the same response.

**Table 4:** Medical sources and vaccine intentions.^a^

Intentions	Number of medical sources followed				
	None	1	2-3	4-5	≥6
Will definitely undergo vaccination, %	28.2	31.7	38.8	55	55.6
Will probably undergo vaccination, %	24.9	23	24.9	17.5	14.8
May or may not undergo vaccination, %	16.6	15.5	19.6	23.8	18.5
Will probably not undergo vaccination, %	14.4	11.7	6.2	0	3.7
Will definitely not undergo vaccination, %	16	18.1	10.5	3.8	7.4

^a^Chi-square test results: *χ*^2^_16_=50.790; *φ*=0.258; *P*<.001.

In contrast, nearly one-third (55/181, 30.4%) of those who did not follow any public health or medical sources said that they would either “probably not” or “definitely not” undergo vaccination. This number fell by roughly 50% among those following at least 2 of the aforementioned sources. A chi-square test showed that the differences were statistically significant (*χ*^2^_16_=50.790; *φ*=0.258; *P*<.001). This observed relationship likely reflects some degree of simultaneity. However, the data did suggest that exposure to credible scientific information on the internet is positively related to compliance with pandemic mitigation measures. Given the high vaccination levels needed to achieve herd immunity, closing even small informational gaps could prove critical to ending the COVID-19 pandemic.

## Discussion

Data from the Pew Research Center show that social media is slowly but steadily supplanting traditional information mediums as a primary source of news and information for many Americans [18]. It has become increasingly clear from existing literature (including our own research) that this trend includes personal and public health information seeking behaviors as well. In this study, we surveyed 1003 American adults on their use of social media to learn about the COVID-19 pandemic. The survey responses confirmed that health consumers have relied heavily on social media to stay up to date with and informed about the COVID-19 pandemic. More than three-quarters (762/1003, 76%) of respondents stated that they have relied on social media at least “a little,” and 59.2% (594/1003) of respondents indicated that they read information about COVID-19 on social media at least once per week. The heavy reliance on social media observed among US-based SNS users is consistent with findings from recent research conducted in various international settings, including China [19] and Europe [20].

Our findings also showed that only about one-third of SNS users (365/1003, 36.4%) have fact-checked pandemic-related information with a medical professional, despite the widespread distrust in the accuracy of COVID-19–related information that is shared on social media. Although disconcerting, this observation is consistent with the previous finding that rigorous fact-checking is relatively uncommon on social media [14]. We also found a greater likelihood of undergoing vaccination among those following more credible scientific sources on social media (*χ*^2^_16_=50.790; *φ*=0.258; *P*<.001), suggesting that scientific credibility may be crucial when promoting compliance with public health guidelines. Recent research has suggested that social media has been instrumental in the spread of vaccine hesitancy [21,22], thereby underscoring the need for health professionals and scientific experts to actively engage with patients and health consumers on social networks in order to address common misconceptions about vaccine safety and efficacy.

Our findings highlight the increasing importance of social media in health information seeking and thus highlight its potential value to health professionals as a conduit for personal and public health communications. However, the growing popularity of SNS platforms for health information seeking is not without its potential drawbacks. Among such drawbacks is the noted propensity for SNSs to facilitate the rapid and widespread dissemination of misinformation and disinformation [4,5,23]. Several studies have examined the presence and effects of misinformation related to COVID-19 since the start of the pandemic. One early analysis of pandemic-related messaging on Twitter suggested that as much as 25% of COVID-19–related information that is being circulated on the platform may contain some degree of misinformation [9]. Recent studies have found that exposure to misinformation on the internet is linked to decreases in the awareness of and compliance with preventative and mitigation measures [24,25]. Although the anonymous and instantaneous nature of social networks can contribute to the rapid spread of health-related misinformation, some research has suggested that social media may also offer an effective avenue for health professionals to counter speculation and misinformation. For example, in a recent experimental study, corrective infographics circulated by the World Health Organization were found to reduce scientific misperceptions about COVID-19 prevention [26].

Another potential drawback of people’s increasing reliance on SNS platforms is the potential for social media overload to increase anxiety and adversely impact the psychological well-being of patients and SNS users. Several recent studies have documented this propensity during the COVID-19 pandemic, though these concerns are likely to be germane to any sustained public health or emergency scenario. One study of young SNS users in the United Kingdom found that information overload related to the COVID-19 pandemic resulted in diminished psychological well-being, including unhealthy levels of the fear of COVID-19 [27]. A similar study that was conducted in Hong Kong during 2020 found a correlation between social media usage and pandemic-related anxiety as well as diminished social trust in information [28]. Although our results showed an increased reliance on social media for health information seeking, these previous findings have suggested that this trend may have adverse mental health impacts for some SNS users—a fact that health professionals will need to be increasingly cognizant of when considering best practices for health communications.

When put into context with the emerging body of literature, our findings suggest that health professionals will need to become increasingly savvy when it comes to social media usage—not just reactively (ie, “setting the record straight”) but also proactively. Given the fact that 76% (762/1003) of health consumers in this survey have relied on social media at least “a little” as a source of health information during the pandemic, accurate and consistent messaging by credible public health organizations is just a start. Based on our research, we believe that this will require more active engagement between health professionals and patients and consumers. To achieve this degree of engagement, health professionals and public health organizations will need to cultivate and customize state-of-the-art social engineering skills to include data mining and natural language processing skills as well as skills that can only be called “active measures” (ie, those for monitoring, anticipating, and responding to misinformation and disinformation on social media platforms), especially during a public health crisis [29]. Furthermore, future research should explore recommendations for institutional policies regarding social media use by government and public health institutions [30].

At the patient level, given the apparent reticence of many social media users to connect with health professionals when fact-checking web-based information, it may be necessary for providers to more deliberately engage patients in conversations about the medical information that they are encountering on social media. Along with raising these issues individually in clinical settings, health care providers can also leverage the evolving functionality of platforms such as Twitter and Facebook to organize live question-and-answer sessions or fact-checking sessions for their patients and communities. For example, during the Zika virus outbreak, the US Department of Health and Human Services held digital town halls via social media. These were routinely advertised through posts such as the following:

Don't miss the @HHSGov #AtoZika Twitter Town Hall tomorrow, Aug. 30, 10 a.m. Submit questions using #AtoZika. #Zika

31

More deliberate networking efforts between local providers and public health agencies may be an effective means of organizing and promoting such events in order to counter misinformation on the internet.

Although these types of broadly targeted communications are critical to effective pandemic management, recent research has also suggested that for many SNS users, personal appeals from reputable practicing physicians may be a more effective means of public health messaging via social media [32]. This type of messaging may be of particular importance in the case of politicized public health emergencies, such as the COVID-19 pandemic. For example, one recent study found that reliance and trust in institutional information sources declined between March and April of 2020 as the pandemic became increasingly politicized [33]. However, our findings suggest that even during the COVID-19 pandemic, a notably low number of Americans have chosen to follow their own clinical providers on social media. Although this may be due to personal choice, the lack of a health care provider presence on social media could be a factor of that choice. For example, a prior survey of physicians indicated that confidentiality, organizational support, and time are all significant barriers to social media adoption [30]. Physicians, physician assistants, and nurse practitioners may need more training on how to effectively use social media to engage with and inform their patient populations. Among other things, such training should address health care providers’ concerns regarding governance, ethics, trust, and patient privacy [34,35]. Notably, prominent organizations such as the Mayo Clinic have already begun incorporating social media literacy into the academic training of health professionals [36].

It is important to acknowledge that adding social media–related duties to the duties of an already overextended health care workforce may further exacerbate the burnout that is experienced by many care providers [37]. However, these data underscore the increasing tendency of patients and consumers to rely on SNS platforms for health-related information. We believe that effective training, institutional support, and proactive collaboration can help health professionals adapt to the evolving patterns of health information seeking behaviors while also protecting the well-being of providers, especially in the midst of an already taxing pandemic.

## Abbreviations

ACS: American Community Survey
SNS: social networking site
